# The association of nationwide trainee doctor mass resignations in South Korea with hospital utilization, expenditure, and patient outcomes

**DOI:** 10.3389/fpubh.2025.1718795

**Published:** 2025-12-12

**Authors:** Seonghoon Kim, SangNam Ahn, Kanghyock Koh, Donghyoun Lee

**Affiliations:** 1 Singapore Management University, Singapore, Singapore; 2 Saint Louis University, St. Louis, MO, United States; 3 Korea University, Seongbuk-gu, Republic of Korea; 4 Jeju National University Hospital, Jeju National University College of Medicine, Jeju-si, Republic of Korea

**Keywords:** South Korea, doctor resignation, medical workforce shortage, hospital, mortality

## Abstract

In February 2024, over 90% of trainee doctors in South Korea’s teaching hospitals resigned en masse, severely disrupting healthcare delivery. We evaluated the associations of this mass resignation with hospital admission and length of stay, expenditure, and patients’ health outcomes in a tertiary teaching hospital. We analyzed inpatient claims data from Jeju National University Hospital (August 2022–July 2024), encompassing 40,577 hospital episodes. Using a difference-in-differences method, we compared outcomes 6 months before and after the mass resignation (February–July 2024 vs. August 2023–January 2024) against the same period 1 year earlier (February–July 2023 vs. August 2022–January 2023) to account for seasonal trends. Primary outcomes included daily hospital admissions, length of stay, total medical costs, 30-day readmission rates, and in-hospital mortality. Analyses were stratified by comorbidity burden using the Charlson Comorbidity Index. The mass resignation was associated with reduced daily hospital admissions (−9.45; 95% CI: −16.1 to −2.77; *p* = 0.01), decreased length of stay (−1.01 days; 95% CI: −1.97 to −0.06; *p* = 0.04), and lower healthcare expenditure (−40%; 95% CI: −68% to −12%; *p* = 0.01). No significant changes were observed in 30-day readmission rates or in-hospital mortality in the overall sample. However, among patients with higher comorbidity burden (CCI ≥ 1), 30-day readmission rates decreased significantly (−0.05; 95% CI: −0.14 to 0.04; *p* = 0.01). Despite no observed short-term mortality increases, reduced utilization among high-comorbidity patients raises concerns about care access during the crisis. The findings suggest that hospitals exhibited short-term adaptive capacity under workforce shortages, while reduced utilization among high-comorbidity patients raises concern for care access. Strengthening contingency planning, flexible staffing, and real-time monitoring systems will be critical to sustaining care quality during future disruptions. Long-term implications of sustained healthcare disruption warrant further investigation.

## Introduction

In February 2024, the government decided to increase medical school admission quotas by 2,000 seats per year (a 67% increase) to improve citizens’ access to healthcare (1–3). However, physicians fiercely opposed the policy announcement, and in protest, more than 90% of trainee doctors (interns, residents, and fellows) from teaching hospitals resigned en masse (3). This action caused major disruptions in healthcare delivery, particularly in emergency care and surgeries, with patients facing difficulty accessing hospital beds, delayed treatments for trauma and cancer surgery, and challenges exacerbated by COVID-19 resurgence (3–6).

The Korean healthcare system, characterized by its single-payer National Health Insurance Service and fee-for-service model, has achieved remarkable improvements in population health over recent decades. Life expectancy increased from 66.1 years in 1980 to 83.6 years in 2021, surpassing the OECD average of 80.3 years, by allocating 9.7% of its GDP to healthcare in 2022 (7). However, the system faces a significant healthcare workforce challenge, with only 2.1 physicians per 1,000 population (excluding Korean traditional medicine doctors), making it the lowest among OECD countries where the average is 3.7 per 1,000 (7). In addition, the number of medical students in Korea has been frozen since 2006 despite multiple attempts by the government to increase due to the resistance of the physician community. While the number of doctors per capita does not necessarily reflect healthcare quality (8), the physician shortage, coupled with a rapidly aging society, risks delaying essential healthcare for many patients, especially in rural areas (9).

The physician community argues that this physician shortage stems from its distorted financial incentives, resulting in longer wait times for patients, shorter consultations, and longer working hours for physicians (5). Equally important, low reimbursements for essential medical services, such as pediatric and emergency care, exacerbates the physician shortage problem in these fields by discouraging trainees from entering these fields while encouraging them to choose more lucrative specialties like plastic surgery and dermatology (10, 11). Tertiary hospitals, designed to manage the sickest patients, are further overcrowded due to the lack of a gatekeeping system and lower out-of-pocket burden even for less severe patients.

As of September 2024, most trainee doctors who resigned still vow not to return unless the government rescinds the entire reform proposal. This unprecedented nationwide protest has significantly disrupted healthcare delivery, particularly in teaching hospitals that heavily rely on trainee doctors for essential daily medical practice (3, 12). For example, medical residents have played a crucial role, accounting for 37.8% of the total number of physicians in Korea’s teaching hospitals, and their absence can significantly compromise the delivery and quality of care (13). The severity of the situation prompted the Korean government to raise the national health disaster warning level from “alert” to “serious”—the highest level (14). Each teaching hospital also entered emergency management mode, incurring net losses and asking employees to take unpaid leave (15).

This crisis presents a unique opportunity to examine how healthcare systems respond to sudden, severe, and large-scale disruptions in the medical workforce supply. Yet, few studies have quantitatively examined the short-term system-wide effects of large-scale physician supply shocks. This research fills that gap by: (1) evaluating changes in hospital utilization, expenditure, and quality of care following the mass resignation of trainee doctors; (2) assessing heterogeneous changes based on patients’ comorbidity burden; and (3) considering healthcare and social implications. We hypothesized that the mass resignation would lead to short-term reductions in hospital admissions, length of stay, and expenditure, but no significant deterioration in immediate patient outcomes.

Using retrospective inpatient claims data, we analyze associations of trainee doctors’ mass resignations with patients’ healthcare utilization, expenditures, and health outcomes. This research provides crucial insights into healthcare system responses to workforce disruptions and identifies areas requiring enhanced preparedness and contingency planning.

## Methods

### Data

We obtained inpatient care data from the Jeju National University Hospital (JNUH), a 720-bed facility. This single-center dataset covers all 40,577 hospital episodes occurring between August 2022 and July 2024. JNUH serves as the primary tertiary care center for Jeju Island (population approximately 694,000 as of June 2025), providing a geographically isolated setting that allows clearer evaluation of workforce disruption effects without substantial patient migration to nearby hospitals. Based on JNUH administrative records, as of February 1, 2024, JNUH employed 164 faculty members, 6 fellows, 68 residents, and 16 interns. By May 1, 2024, 67 residents and all 16 interns had resigned, whereas faculty and fellow numbers remained stable at 167. This represented a complete withdrawal of the training physician workforce.

### Outcome measures

Our primary outcomes of interest were (i) number of daily admissions per each month, (ii) hospital length of stay (LOS), (iii) total medical costs charged (in logarithm), (iv) share of 30-day readmission among newly admitted patients, and (v) in-hospital deaths per hospital admission. We measure a patient’s comorbidity burden via the Charlson comorbidity index (CCI) (16). However, our dataset does not contain physiological scores (e.g., APACHE, NEWS) and vital signs. As a result, we could not incorporate these analyses, and CCI-stratified results should be interpreted as reflecting baseline health status and comorbidity burden rather than acute illness severity. Each hospital admission was analyzed as a separate episode. Readmissions were identified based on a 30-day interval following discharge for the same individual.

### Study design and statistical analysis

We employed a difference-in-differences (DID) method to examine associations of the trainee doctors’ mass resignations with the aforementioned outcome measures. We compared outcomes 6 months before and after the mass resignation in February 2024 (treated period: February 2024–July 2024 vs. August 2023–January 2024) against the same period 1 year earlier (control period: February 2023–July 2023 vs. August 2022–Jan 2023). We selected six-month pre- and post-periods to balance data availability and capture sufficient time for hospital adaptation following the February 2024 resignation.

We implemented this research design by running a DID regression of each outcome variable on the post-resignation month dummy (indicating February to July), the treated season dummy (indicating August 2023–July 2024), and its interaction term. We also adjusted for the age and gender of patients in the regression. All analyses were performed in August 2024 using Stata, version 18.5 (Stata Corp).

### Statistical model specification

We use the following DID specification:


$$y_{t}=\beta_{0}+\beta_{1}\mathrm{Pos}t_{t}\times \text{Trea}t_{t}+\beta_{2}\mathrm{Pos}t_{t}+\beta_{3}\text{Trea}t_{t}+X\prime_{t}\gamma +\in_{t},$$

where $y_{t}$ represents outcome variables of interest in a calendar year-month *t*; $\mathrm{Pos}t_{t}$ is a dummy variable equal to 1 for the period from August 2023 to July 2024, and 0 otherwise; $\text{Trea}t_{t}$ is a dummy variable equal to 1 if the observation month is between February and July, and 0 otherwise. X includes the aforementioned control variables including the average patient age and the share of male patients. $\epsilon_{t}$ is an error term. For statistical inference, we calculate heteroskedasticity-robust standard errors. As data come from a single hospital, clustering was not applied.

The coefficient of interest is $\beta_{1}$ , which captures the associations between the mass resignations and outcome variables. To assess the validity of the DID approach, we visually inspected pre-resignation (pre-treatment) trends for all outcomes to verify that the parallel trends assumption held. The plots confirmed stable pre-event trajectories, supporting the DID design.

For log-transformed total inpatient care expenditure, the estimated $\beta_{1}$ is interpreted as an association between the mass resignation and changes in hospital expenditure by 100* $\hat{\beta_{1}}$ %. For hospital readmissions and mortality rates, as described from the Data subsection, we calculate the number of hospital readmissions and in-hospital deaths per hospital admission. Thus, $\beta_{1}$ is interpreted as the number of hospital readmissions or in hospital death per hospital admission. We used linear regression models for all outcomes.

We acknowledge that our observational study design, despite using DID methodology, cannot fully rule out all potential confounding factors. Unobserved contemporaneous changes, such as hospital-specific policy responses, concurrent COVID-19 effects, or patient behavioral changes, could influence outcomes. Therefore, we interpret our findings as associations rather than definitive causal effects.

## Results

Our analysis revealed significant changes in healthcare utilization and outcomes following the mass resignation of junior physicians. Table 1 presents the descriptive statistics for key variables across four time periods. We first observe that the total number of patients hospitalized decreased significantly by 1,597 during the mass resignation period (February to July 2024; Period 4) compared to that a year ago (Period 2), which is 9,104. This decrease could be driven by a seasonal trend in 2024, but the difference between Period 2 and Period 4 is 3.3 times larger than the difference between Period 1 and Period 3 (which is 483).

**Table 1:** Descriptive statistics for key variables across four time periods.

Variable	(1) Period 1	(2) Period 2	(3) Period 3	(4) Period 4	(5) *P*-value
	2022.8–2023.1	2023.2–2023.7	2023.8–2024.1	2024.2–2024.7	
Number of patients	9,186	9,104	8,703	7,507	
Variable	Mean (±SD)	Mean (±SD)	Mean (±SD)	Mean (±SD)	
Age	56.8 (±0.96)	56.8 (±0.8)	56.7 (±1.09)	55.8 (±0.74)	0.167
Share of male	0.53 (±0.01)	0.52 (±0.01)	0.52 (±0.02)	0.52 (±0.02)	0.974
Charlson comorbidity index	0.69 (±0.04)	0.69 (±0.02)	0.68 (±0.03)	0.71 (±0.08)	0.717
Daily number of hospital admissions	65.3 (±3.71)	66.5 (±1.91)	62.2 (±5.18)	54.9 (±1.24)	<0.001
Length of stay	7.96 (±0.44)	7.60 (±0.21)	7.65 (±0.41)	6.21 (±0.92)	<0.001
Total daily inpatient care expenditure (in US$1,000)	456,890 (±64,115)	421,105 (±44,400)	430,147 (±70,348)	264,658 (±55,025)	<0.001
Proportion of 30-day hospital readmissions among newly admitted patients	0.17 (±0.05)	0.19 (±0.001)	0.19 (±0.01)	0.20 (±0.01)	0.183
Number of in-hospital deaths per hospital admission	0.04 (±0.01)	0.03 (±0.001)	0.04 (±0.001)	0.03 (±0.01)	0.178

The mean age of hospitalized patients was about 56 years, and the share of male patients remained consistent at around 52%–53%, indicating relatively stable patient demographics during the study period. However, the mean CCI increased from 0.68 to 0.71 post-resignation between columns (3) and (4), while such an increase was not observed during the same months a year ago between columns (1) and (2). However, none of these patient characteristics were statistically significantly different from one another.

The daily number of hospital admissions decreased from 62.2 (SD 5.18) to 54.9 (SD 1.24) following the mass resignation in February 2024, even when it increased during the same months a year ago (Period 1 vs. Period 2). In addition, the LOS also decreased significantly by 18.8% from 7.65 to 6.21 between 6 months before and after the mass resignation. As a result, total daily expenditure of inpatient care decreased by about 40% from US$430,147 to US$264,658.

Contrary to the decrease in utilization measures, we do not find clear evidence of decreases in health outcomes. For example, the proportion of 30-day hospital readmissions among newly admitted patients ranged 0.17 to 0.20 across the four periods, but the differences were not statistically significant (*p*-value = 0.183). The number of in-hospital deaths per hospital admission even decreased following the mass resignation, albeit statistically insignificant (*p*-value = 0.178).

Table 2 presents the distribution of hospital admissions by clinical department before and after the mass resignation. The overall department composition remained largely stable, though orthopedic and general surgical admissions slightly declined, while pediatric and internal medicine admissions modestly increased, consistent with hospitals prioritizing essential and acute care during the disruption.

**Table 2:** Distribution of hospital admissions by clinical department before and after trainee doctor mass resignation.

Department	Before resignation (August 2022–January 2024)	After resignation (February–July 2024)
Internal Medicine	40.4%	41.1%
Orthopedic Surgery	11.8%	9.9%
General or Thoracic Surgery	9.1%	8.5%
Pediatrics	7.6%	9.0%
Obstetrics and Gynecology	7.5%	7.3%
Ophthalmology	6.5%	6.9%
Neurology or Neurosurgery	5.0%	5.1%
Other	4.7%	3.3%
Urology	4.1%	5.0%
Otorhinolaryngology	3.4%	3.8%

**Figure 1:**
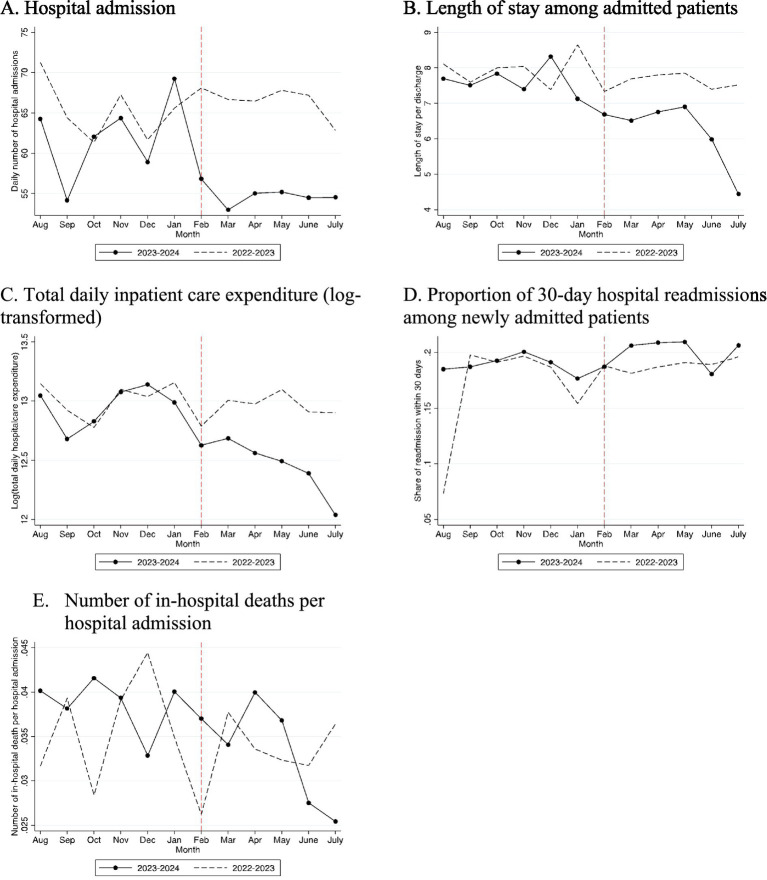
Graphic presentation of hospital admission, length of stay, healthcare expenditure, 30-day hospital readmissions, and in-hospital deaths (unadjusted). **(A)** Hospital admission, **(B)** length of stay among admitted patients, **(C)** total daily inpatient care expenditure (log transformed), **(D)** proportion of 30-day hospital readmissions among newly admitted patients, and **(E)** number of in-hospital deaths per hospital admission.

Figure 1 presents unadjusted monthly trends in hospital admission and other key outcomes. It provides consistent evidence of those in Table 1. Panels A to C indicate that hospital admissions, LOS, and healthcare expenditure sharply decreased after the mass resignation in February 2024, compared to the same period a year ago. These reductions likely reflect selective deferral of elective or non-urgent admissions and resource reallocation to sustain emergency and high-severity cases, as teaching hospitals prioritized limited workforce capacity. Despite lower utilization, Panels D and E document little evidence that 30-day hospital readmission rates and in-hospital mortality rates do not appear to increase after the mass resignation.

Then we formally estimate these associations between the mass resignation and hospital outcomes, using our DID method. Table 3 shows DID estimates, which quantify the associations of the mass resignation with hospital outcomes after adjusting for underlying time trends. In Panel A, we use the entire sample. Column (1) shows a significant reduction of 9.45 admissions per day (95% CI, −16.1 to −2.77; *p* = 0.01) attributable to the resignation event. Columns (2) and (3) indicate that LOS decreased by −1.01 (95% CI, −1.97 to −0.06; *p* = 0.04) and total healthcare expenditure decreased by 40% (95% CI, −0.68 to −0.12; *p* = 0.01). However, we did not find evidence of adverse health impacts measured by 30-day readmission and in-hospital deaths in columns (4) and (5). Because all resident physicians and interns departed while faculty staffing remained essentially unchanged, the observed reductions in hospital utilization occurred despite the hospital maintaining approximately 67% of its pre-resignation physician workforce.

**Table 3:** Associations between mass resignation and hospital admissions, length of stay, hospital expenditure, 30-day hospital readmissions, and in-hospital death from August 2022 to July 2024: a difference-in-differences analysis.

Dependent variable	Hospital Admission	Length of stay	Log (total inpatient care expenditure in US$1,000)	30-day hospital readmissions	In-hospital death
	(1)	(2)	(3)	(4)	(5)
A. The entire sample					
Treat × Post	−9.45	−1.01	−0.4	−0.001	−0.001
95% Confidence Interval	−16.1, −2.77	−1.97, −0.06	−0.68, −0.12	−0.04, 0.03	−0.01, 0.01
*p*-value	0.01	0.04	0.01	0.91	0.88
B. Patients with a Charlson comorbidity index of zero					
Treat × Post	−7.14	−0.77	−0.26	0.001	−0.001
95% Confidence Interval	−13.22, −1.06	−1.83, 0.29	−0.62, 0.11	−0.02, 0.02	−0.01, 0.01
*p*-value	0.02	0.14	0.15	0.87	0.69
C. Patients with a Charlson comorbidity index of one or higher					
Treat × Post	−2.27	−1.59	−0.57	−0.05	−0.001
95% Confidence Interval	−4.97, 0.44	−2.87, −0.31	−1.00, −0.14	−0.14, 0.04	−0.02, 0.01
*p*-value	0.1	0.1	0.02	0.01	0.28

Panel B restricted the sample to patients with the CCI of zero. Column (1) presents that the resignation was associated with a 7.14 decrease of hospital admissions per day (95% CI, −13.2 to −1.06; *p* = 0.02). Columns (2)–(5) indicate that the associations of the resignation with LOS, total inpatient care expenditure, 30-day readmission, and in-hospital deaths were not statistically significant.

Panel C restricted the sample to patients with the CCI of one or higher. Column (1) presents that the resignation was associated with a 2.27 decrease of hospital admissions per day (95% CI, −4.97 to 0.44; *p* = 0.10). Columns (2) and (3) indicate that LOS decreased by −1.59 (95% CI, −2.87 to −0.31; p = 0.10) and total healthcare expenditure decreased by 57% (95% CI, −1.00 to −0.14; p = 0.10). Importantly, 30-day readmission rates decreased by 0.05 per admission (95% CI: −0.14 to 0.04; *p* = 0.01), likely reflecting barriers to accessing necessary follow-up care during the crisis rather than improved outcomes. In-hospital mortality did not change significantly (*p* = 0.28).

Because our analysis relies on an observational DID design, the results should be interpreted as associations rather than causal effects, as concurrent hospital or patient-level responses may have contributed to the observed changes.

## Discussion

This study revealed that the mass resignation of trainee doctors at a teaching university hospital in Korea was associated with lower hospital utilization, including reduced hospital admissions and length of stay, as well as decreased hospital expenditure, without significant changes in in-hospital mortality rates. These associations varied by patients’ comorbidity burden (CCI). We observed a decrease in 30-day readmission rates among patients with higher comorbidity burden (CCI ≥ 1), which likely reflects restricted access to follow-up care or incomplete transitions of care rather than improved outcomes.

Our study contributes to the growing body of literature on healthcare worker strikes and their impacts on health systems. Globally, healthcare workers, frustrated with unfavorable working conditions and compensation, have gone on strike in various countries, including Nigeria, Spain, the United Kingdom, New Zealand, and the US (17–22). Most prior studies found significant reductions in patient volumes and readmissions without reporting increased mortality rates during the strikes (17–23). However, the previous physician strikes were relatively smaller scale and short-run. The prolonged nature and extensive scale of the current seven-month-long crisis in Korea may lead to more severe and long-lasting consequences. Since February 2024, the situation has worsened as the government and trainee doctors remain uncooperative and hostile toward each other and most trainee doctors who left in February still have not returned as of September 2024 (24); to make matters worse, the recent spread of COVID-19 is straining the already burdened Korean healthcare system (6).

The observed 40% reduction in hospital expenditure (Table 3, Column 3) raises significant concerns about the financial stability of tertiary hospitals that heavily rely on trainee physicians. This financial strain could potentially trigger potential longer-term impacts throughout the Korean healthcare and medical industry, affecting not only hospitals but also medical device manufacturers and pharmaceutical companies. These industry-wide changes could significantly worsen citizens’ health.

We found no significant changes in overall 30-day readmission or in-hospital mortality rates following the mass resignation. However, this apparent stability could conceal important differential impacts across patient groups. We observed that reductions in hospital length of stay and expenditure were more pronounced among patients with higher CCI scores. While our data do not include information on bed occupancy, procedure volumes, or clinical interventions, several mechanisms could explain these patterns. First, hospitals may have shortened or compressed care episodes to maintain throughput for urgent cases during the workforce disruption. Second, stricter triage may have limited admissions to the most acute patients while deferring less-urgent or chronic cases. Third, access constraints could have led to deferred admissions for chronic disease management, which normally require prolonged stays. Together, these potential mechanisms suggest that observed declines in utilization among sicker patients do not necessarily represent efficiency gains but could indicate rationing of care or unaddressed healthcare needs.

Importantly, the absence of a detectable increase in in-hospital mortality over the six-month observation window should not be interpreted as evidence of preserved care quality or system resilience. Short-term survival may mask latent deterioration in patient outcomes that emerges after discharge or through foregone hospitalizations. The findings therefore highlight a critical distinction between apparent resilience in immediate outcomes and the possibility of delayed health consequences. Longer follow-up using multi-hospital data is essential to determine whether these short-term adaptations represent genuine resilience or constrained access that could compromise long-term patient well-being.

Our study has several limitations that warrant consideration. First, its focus on a single university teaching hospital on an island with a population of around 677,000 may limit generalizability. Our single-site data may not fully capture patients who delayed, diverted, or forewent care during the mass resignation. This introduces potential selection bias in patient presentation and outcomes. Larger hospitals in metropolitan Seoul tend to hire more residents and attract higher-risk patients (13). Thus, the negative health impacts of the mass resignation could be more pronounced in larger hospitals. Second, our dataset does not include workforce composition data, such as the actual number of residents before and after the mass resignation and replacement staffing patterns. Thus, our findings should be interpreted as associations between the resignation event and hospital outcomes rather than direct causal estimates of specific staffing level changes. Third, our analysis is limited to the immediate six-month impact due to data availability. The full health consequences of the current crisis, particularly those resulting from delayed or forgone treatments, may not have materialized within this time frame. Fourth, this study does not examine the effects on hospitals’ profits, which could affect their long-term resilience and adaptability, due to the lack of data. Finally, although our difference-in-differences design accounts for seasonal patterns, unobserved contemporaneous shocks, e.g., policy responses or local COVID-19 surges, may confound estimates; results should thus be interpreted as associations rather than causal effects.

Future research should focus on the longer-term impacts of this crisis, including potential disparities in care access, psychological impacts on patients and healthcare workers, and the effectiveness of policy interventions. Additionally, future national-level studies using linked administrative datasets will be crucial to assess long-term and broader system-wide impacts.

In conclusion, this study offers valuable insights into the short-term associations of severe disruptions in the physician workforce with hospital utilization, hospital expenditure, and patient outcomes in Korea. We found a significant reduction in inpatient care volumes and hospital expenditures among patients with both mild and severe disease profiles. We also raised concerns about the potential long-term compromise in the quality of patient care. From a policy perspective, the evidence underscores the importance of developing flexible workforce deployment systems and real-time national monitoring of hospital capacity to mitigate the effects of future healthcare labor disruptions. Targeted incentives to retain trainees in essential specialties and strengthen inter-hospital referral coordination may enhance system resilience during prolonged crises. Finally, concerted efforts should focus on rebuilding trust among key stakeholders to restore collaboration and ensure sustainable, high-quality care delivery.

## Data Availability

The raw data supporting the conclusions of this article will be made available by the authors, without undue reservation.
